# What is the level of uptake of partner notification services in HIV testing in selected health facilities in Gatanga Sub County, Muranga County – Kenya; a retrospective study

**DOI:** 10.1186/s12879-020-05146-9

**Published:** 2020-06-22

**Authors:** Rewel Mwangi Kariuki, Gilbert Koome Rithaa, Elvis Omondi Oyugi, Daniel Muya Gachathi

**Affiliations:** 1 Department of Health, Murang’a County Government, P O Box 69, Muranga, 10200 Kenya; 2 College of Health Sciences, Mount Kenya University, P O Box 342, Thika, 01000 Kenya; 3 Field Epidemiology & Laboratory Training Program (FELTP)–Kenya, P. O Box 30016, Nairobi, Kenya

**Keywords:** Partner notification services, HIV, Contact, Kenya

## Abstract

**Background:**

Identification of people living with HIV is key in HIV prevention and control. Partner Notification service is a World Health Organization backed strategy of reaching out to sexual partners of people diagnosed with HIV for HIV testing. However, its adoption and success rate in Kenya remains unknown.

**Methods:**

A cross sectional facility based study was undertaken in five purposely selected health facilities in Gatanga Sub county, Muranga County, Kenya. A retrospective review of patient medical records data for HIV positive index clients and their Sexual Partners conducted. Census approach was applied to extract data for study subjects from Partner Notification Services registers for the period covering January 2017 to August 2018. Epi Info software was used for data analysis.

**Results:**

A total of 183 index clients were offered Partner notification services. The mean age of the indexed clients studied was 39(SD ±13.1). Females comprised 64% of clients studied. Of the 183 indexed clients, 89% accepted the services and elicited 216 sexual partners for tracing. The ratio of elicited sexual partners to index client was 1.3:1. Out of the 216 sexual partners, 77% were reached and tested. A total of 46 [32%] of the sexual partners elicited and traced, tested HIV positive. The most preferred approaches were provider referral (51%) and contract referral (45%). Dual referral (4%) was the least preferred approach.

**Conclusions:**

Assisted Partner notification services is acceptable and an effective strategy of increasing HIV case identification and raising awareness to exposed sexual partners in low resource countries.

## Key messages

- Partner notification services is an effective strategy for HIV case identification in developing countries where HIV uptake is low.
- Provider referral is the most preferred approach of contacting sexual partners for HIV testing; however, the contract referral approach had the highest success rate of reaching and testing the sexual partners.
- PNS is most effective when reach out messages are provided in more than one attempt; Only half of sexual partners are successfully reached in subsequent attempts after initial contact is made.

## Background

The HIV/AIDs epidemic remains among the greatest public health concerns globally with approximately 37.9 million people living with Human Immunodeficiency Virus (HIV) in 2018. Of these 79% knew their HIV status [1]. Eastern and Southern Africa carries the highest burden of HIV with half of the world’s people living with HIV. The region contributed 46% of the world’s new HIV infections in 2015 [2].

Kenya is one of the four countries in Africa with a high burden of HIV with a prevalence of 4.9%. This is a decline from 5.5% in 2014. HIV burden is variably distributed geographically across Kenya and thus requires intense efforts to reduce the burden. There were approximately 52,800 new infections across all ages in Kenya in 2017 where 44,800 were adults and 8000 were children aged < 14 yrs. There is a gradual decline in adult HIV incidence from 0.27% in 2016 to an estimated 0.19% in 2018 with young women aged 15-24 yrs. accounting for a third of all new HIV adult infections [3, 4].

Murang’a County is one of the 47 counties in Kenya. It has seven sub counties and an estimated population of 1,053,059 [5]. The estimated number of people living with HIV in Murang’a County is 30,376. By the year 2017, only 13,857 people (45.6%) had been identified and were receiving care and treatment. This is far below the national coverage of 75% and far off the UNAIDS 909090 targets [3]. Gatanga sub county is the second largest sub county in Murang’a County with a total population of 187,601 [5]. Its divided into two regions, upper and lower Gatanga. In 2017, a total of 356 people were newly diagnosed with HIV in Gatanga sub county. By December 2017 there were a total of 1952 HIV patients in care. To continue reducing the number of new HIV incidence, scaling up of prevention interventions is paramount with an aim at increasing awareness and change in behaviour. HIV Partner Notification Services (PNS) is a strategy in HIV prevention programs where a trained provider requests people diagnosed with HIV to voluntarily provide information about their sexual partners and/or drug injecting partners and efforts are made to reach them for HIV testing [6].

Partner services are underutilized in Kenya therefore more research on the coverage and implementation gaps for HIV Partner Notification services is required. In Murang’a County, Partner Notification Services strategy has been adopted as part of routine HIV testing services (HTS) and is being implemented in all health care facilities where HIV testing services are offered by HTS counsellors. In Gatanga sub county there are 41 health facilities, of which 14 of them offer assisted partner notification services. There is limited evidence on uptake and effectiveness of use of PNS in sexual partner elicitation, notification and testing in Kenya. The aim of this study findings was to support policy makers and health managers on improving implementation of PNS services aimed at achieving increasing HIV case identification and coverage of care and treatment.

## Methods

The study was a retrospective; health facility based study-involving review of secondary data from PNS registers. The registers documented HIV positive index clients who were offered PNS and the sexual partners elicited. The review period covered January 2017 to August 2018. In this period, a total of 273 clients were offered assisted partner notification services in Gatanga sub county. Five out of 14 health facilities implementing PNS in Gatanga sub county were purposively selected for review based on the high volume client workload and availability of HTS counsellors to provide the services. Only clients offered PNS in the five sampled facilities were eligible in this study. The selected facilities included one sub-county hospital, two health centres (each from lower and upper Gatanga) and two dispensaries (each from lower and upper Gatanga) as shown in Table 1 below.

**Table 1:** Table showing selected facilities, region and number of records

Facility	Sub county Region	Index clients with complete records	Index clients with incomplete records	Total Index Clients Records
Kirwara Sc Hosp	**Upper Gatanga**	69	6	75
Gatura Hc	**Upper Gatanga**	22	3	25
Gatunyu Dispensary	**Upper Gatanga**	36	2	38
Ithanga Hc	**Lower Gatanga**	47	2	49
Ngelelya Dispensary	**Lower Gatanga**	9	6	15
Total		183 **(90.5%)**	19 **(9.5%)**	202 **(100%)**

Enrolment of clients to the study was also delimited to availability and completeness of data in the PNS registers from the 5 health facilities meeting the criteria; that is, indexed clients and sexual partners elicited. An incomplete record was defined to be any record missing any of the key analytical variables which included; Age, sex, PNS accepted, and date tested. Census approach was used to list and enrol 183 index study subjects whose records were complete.

In practice, assisted partner notification services (aPNS) is offered to a HIV positive index client only where express consent of the client has been granted and should only be to their partners alone. It involves health care providers encouraging a HIV-positive client (index case) to voluntarily disclose information of past and present sexual partner(s). Details of the index client and their sexual partners are recorded in the PNS register. Upon successful elicitation of sexual partners, the provider explains to the client the available approaches which can be used to reach the sexual partners where the client chooses the preferred method for informing each partner.

The following are the different approaches;

i. Contract referral; This is where a HIV positive client enters into a mutual agreement with a health care provider to refer their partner for testing within an agreed time period after which the provider contacts the partner(s) directly for testing.
ii. Provider referral; Where a health care provider with the consent of the HIV positive client contacts the partners elicited directly for HIV testing.
iii. Dual referral; When a health care provider together with an HIV-positive client makes efforts to reach to partner(s) for HIV testing.

Follow up to reach the sexual partners was done through phone calls and text messages.

In this study, patient records data was abstracted using two data abstraction tools and entered into an excel sheet from the PNS registers; the first checklist contained details of all the index clients and the second checklist contained details on all the sexual partners elicited. The checklist comprised a number of study variables comprising facility code, subject code, residence, occupation, age, relationship to client, currently living with index client, marital status of the index client and intimate partner violence. Other variables were index client modality, knowledge of HIV status, preferred PNS approach, PNS accepted [y/n], partner reached [y/n], number of attempts to reach, consent to testing [y/n], tested [y/n] and HIV test results [pos/neg/i/na].

Epi-Info, a data analytic software was used to clean the data and perform descriptive statistics for the study. Study authorization and permission sought from relevant institutions. Dummy codes [anonymised] assigned to facilities and study subjects. Password protected databases were also applied as part of confidentiality measures.

## Results

Records of 183 index clients in the period between January 2017 to August 2018 reviewed. Mean age for the index clients was 39 [SD ± 13.1] and most were female 118 [65%]. Table 2 below shows socio-demographic characteristics for the 183 index clients and 216 sexual partners elicited. Majority of clients were aged between 25 and 34 years. Most of the index clients 100 [55%] were in monogamous marriage while only 4 [2%] reported to be in polygamous marriage. Youth and teenagers aged 24 years and below were 13% [23] among the index clients and 15% [31] among the sexual partners elicited.

**Table 2:** Socio-demographic characteristics of index clients and sexual partners

		Index Clients		Sexual Partners	
		Frequency	Percent	Frequency	Percent
Sex	Male	65	36%	119	55%
	Female	118	64%	97	45%
	**Total**	**183**	**100%**	**216**	**100%**
Age Category in Years	≤24	23	13%	31	15%
	25–34	56	31%	85	39%
	35–45	52	28%	55	25%
	46+	52	28%	45	21%
	**Total**	**183**	**100%**	**216**	**100%**
Occupation	Formal Employment	16	9%	5	2%
	Informal Employment	45	25%	64	30%
	Not employed	26	14%	25	12%
	Self employed	96	52%	122	56%
	**Total**	**183**	**100%**	**216**	**100%**
Marital Status	Divorced/Separated	28	15%	No data^a^	No data^a^
	Married monogamous	100	55%		
	Married polygamous	4	2%		
	Single	41	22%		
	Widowed	10	6%		
	**Total**	**183**	**100%**		

^a^The PNS registers did not capture the marital status of the sexual partners

### PNS acceptance

**Fig. 1:**
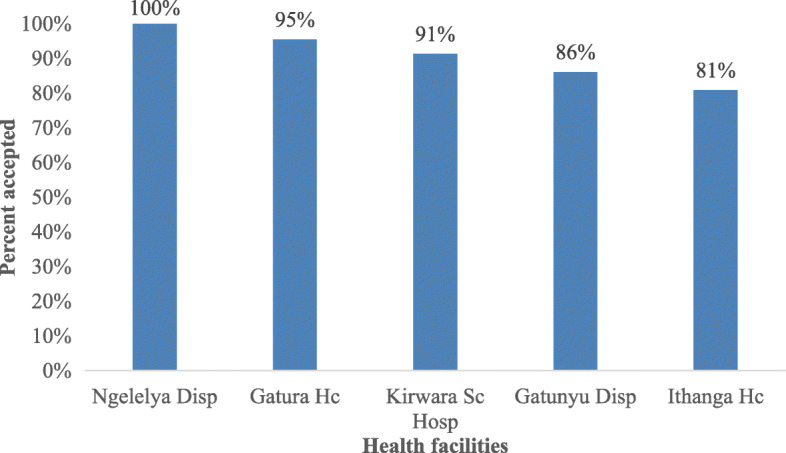
PNS acceptance by health facility

Overall, 162 [89%] index clients accepted PNS. Acceptance rate was higher among male index clients [92%] than in female [86%]. The rate of acceptance per health facility slightly varied with Ngelelya dispensary having a 100% acceptance rate and the lowest was Ithanga health centre, 81% as shown in Fig. 1 below. Of the 21 index clients who did not accept PNS, most were female 16 [76%]. Acceptance rate was highest among index clients aged 25-34 yrs. [56,93%] with the lowest being among index clients aged 35-45 yrs. [43,83%].

**Fig. 2:**
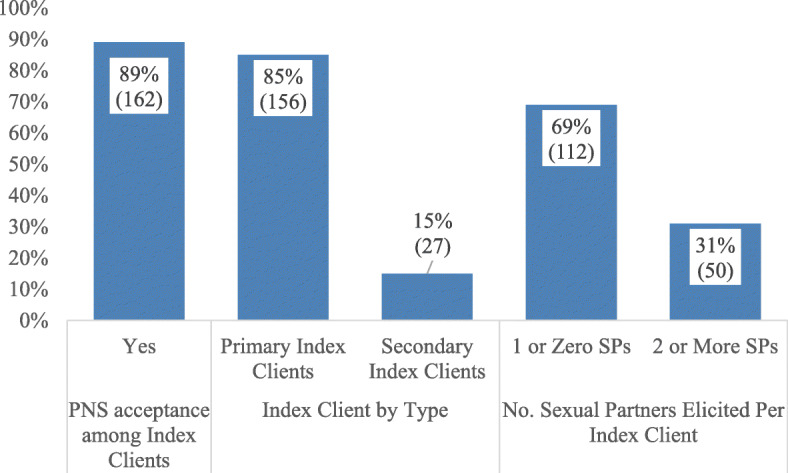
Indexed Clients by sex, PNS acceptance, and number of sexual partners elicited

Among the index clients who accepted PNS, 50 [31%] elicited two or more sexual partners and 27 [15%] of the total index clients were secondary index clients. {Secondary Index client is one who had been listed by another index client} (see Fig. 2).

### PNS approach and relationship status

**Fig. 3:**
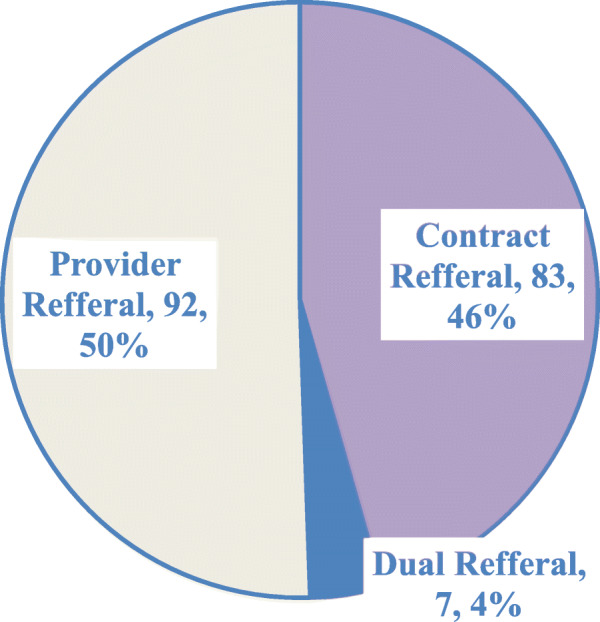
PNS approach as preferred by index clients [*n* = 182]

The most preferred approach of contacting and tracing the sexual partners was Provider referral [92, 50%], and the least preferred was Dual referral [7, 4%] as shown in Fig. 3 below. Only 50% of SPs followed through dual referral approaches were successfully reached and tested followed by provider referral approaches where 84% were successfully reached and tested. Contract referral approach recorded the highest success rate in reaching SPs where 97% were successfully reached.

Majority of index clients reported they were not currently living with their listed sexual partners [154, 57%]. While only 15% [34] of sexual partners had their HIV status known by their index clients, for most of the sexual partners [182, 85%] HIV status was unknown to the index clients.

### Elicitation of sexual partners

Among male index clients 25 [42%] listed two or more sexual partners which is higher than that of female index clients where only 25 [25%] listed two or more sexual partners as shown in Table 3 below. For index clients aged below 25 years 9 [43%] elicited 2 or more sexual partners while among index clients with formal employment 56% elicited 2 or more sexual partners.

**Table 3:** Table showing demographics of index clients by number of partners elicited

		Index clients who elicited one or Zero Sexual partner		Index clients who elicited 2 or more Sexual partners		
		No.	Percent	No.	Percent	Total
**Age Category**	≤24	12	57%	9	43%	21
	25–34	38	66%	20	34%	58
	35–45	22	59%	15	41%	37
	46+	40	87%	6	13%	46
**Sex**	Female	77	75%	25	25%	102
	Male	35	58%	25	42%	60
**Marital Status**	Divorced/Separated	17	71%	7	29%	24
	Married monogamous	68	72%	27	28%	95
	Married polygamous	2	50%	2	50%	4
	Single	21	64%	12	36%	33
	Windowed	4	67%	2	33%	6
**Employment Type**	Formal Employment	7	44%	9	56%	16
	Informal Employment	27	64%	15	36%	42
	Not Employed	15	83%	3	17%	18
	Self employed	63	73%	23	27%	86

### Outcome for sexual partner tracing

For the 172 SPs whose attempts to reach them were made, 157 [91%] were successfully reached. Of the sexual partners where only one attempt had been done, 99% were reached and the rate reduced with more attempts to reach the sexual partners where only 70% of the sexual partners with three attempts made to reach them were successfully reached (Table 4 below).

**Table 4:** Table showing number of sexual partners reached and number of attempts made [*n* = 172]

Attempts to reach sexual partners	Sexual partners reached [%]	Sexual partners not reached [%]
First attempt	**91 (53%)**	**1 (1%)**
Second attempt	**51 (30%)**	**8 (3%)**
Third attempt	**14 (8%)**	**6 (9%)**
Total	**157 (91%)**	**15 (9%)**

Ngelelya dispensary had the highest positivity rate of 59% followed by Ithanga Hc with 49% and the lowest positivity rate was reported in Gatura Hc [10%] (Table 5 below).

**Table 5:** Table showing outcomes of sexual partners tracing and HIV testing per health facility

Health facility	Index clients accepted PNS	Sexual partners elicited	Ratio of partners to index clients	Sexual partners eligible for testing	HIV tested	Positivity rate
Kirwara	63 (91%)	89	1.41	65	44 (68%)	11 (25%)
Ithanga	38 (81%)	37	0.97	37	37 (100%)	18 (49%)
Gatunyu	31 (86%)	29	0.94	21	13 (62%)	4 (31%)
Gatura	21 (95%)	44	2.10	42	30 (71%)	3 (10%)
Ngelelya	9 (100%)	17	1.89	17	17(100%)	10(59%)
**Total**	**162 (89%)**	**216**	**1.33**	**182**	**141 (77%)**	**46 (32.6%)**

The ratio of sexual partners to index clients varied across the health facilities where Gatura had the highest ratio of 2.10:1. Overall ratio of sexual partners to index clients was 1.33:1 as shown in Table 4 below. Out of 182 sexual partners eligible for HIV testing, 141 [77%] were successfully tested with 46 [32.6%] identified as HIV positive.

## Discussion

These study findings demonstrate that PNS is generally acceptable and effective in increasing HIV case identification. The findings of this study on PNS acceptance shows a higher rate of PNS uptake than that of another RCT done in Kenya where acceptance rate was 67% [7]. Routine implementation of the PNS as part of HTS in health facilities would contribute to increased HIV case finding. The ratio of sexual partners to index clients varied significantly across gender and health facilities. The strategy was most effective in eliciting male SPs. This is similar to a study done in Tanzania where male index clients were found to be 6.2 times more likely to list more than one sexual partner [8]. There were more female index clients than male hence the high rate of male sexual partners’ elicitation. Further, uptake of voluntary HTS was found to be higher among female sexual partners compared to males.

PNS shows potential benefits for increasing HIV case identification as well as increasing awareness of possible exposure to HIV among SPs [7]. Undertaking more than one follow up to reach the sexual partners increases the potential for successful elicitation.

HIV testing uptake among partners elicited varied across facilities with lowest uptake being 62% and highest being 100%. Findings showed higher uptake in Sub-county hospital [Kirwara] compared to other lower facilities. While this can be attributed to better training and elicitation skill set among HTS in the higher facilities compared to lower facilities [9], there is low level of evidence to support this explanation. A Kenyan study on barriers to scale up of PNS seemed to support this explanation [9]. The study acknowledged need for capacity building of health workers to support delivery of effective PNS through improved elicitation and client engagement skills. In Kenya, PNS has just been adopted as part of comprehensive HIV testing services; there are no robust studies to explain possibility of differences in elicitation skills among different cadres of HCWs. This is a gap which needs to be considered for future studies.

The study established the first time contact acceptance rate of PNS among clients to be higher compared to that of index clients previously identified. This is similar to findings in a study in Tanzania on outcomes and experiences of men and women with partner notification for HIV testing [8]. Higher first-time acceptance rate was linked to higher volume of elicited clients who are willing and ready to know their status compared to other contact stages. In addition, after first contact, clients may choose to seek HIV testing in other facilities while others may already be knowing their status. An RCT done in Kenya also showed similar findings where immediate PNS significantly increased partner HIV testing compared to delayed group [10].

## Conclusions

Partner notification services is an effective strategy of increasing HIV case identification in high prevalence areas such as Sub Saharan Africa. With many people engaging in sex with HIV positive individuals, increasing vulnerability to HIV infection, PNS has demonstrated to be a better way of reaching those exposed for testing and thus increase potential for protection against infection with HIV. To achieve the ambitious 909090 targets as stipulated by WHO in the fight against HIV/AIDs, adoption of PNS in routine HTS is worth and will accelerate achievement of first 90, which is the entry point to care and treatment for HIV positive people. PNS is acceptable and implementable in all levels of health facilities.

### Limitations of the study

These study results have some shortcomings. Considering the study used secondary data, there were some incomplete data such as on marital status for sexual partners, which was not documented. The registers also do not give details on how follow ups was done.

## Data Availability

The dataset used for this study is available from the corresponding author on reasonable request.

## Abbreviations

AIDS: Acquired Immune Deficiency Syndrome
FELTP: Field Epidemiology & Laboratory Training Program
HIV: Human Immunodeficiency Virus
PNS: Partner Notification Services
HTS: HIV Testing Services
MoH: Ministry of Health
aPNS: Assisted Partner Notification Services
RCT: Randomized Control Trials
SPs: Sexual Partners (SPs)
UNAIDS: United Nations Programme on HIV and AIDS
WHO: World Health Organization
